# Depth profiling X-ray photoelectron spectroscopy and atomic force microscopy of Cd(ii)- and Pb(ii)-selective electrodes based on nano metal sulfides

**DOI:** 10.1039/c7ra13168b

**Published:** 2018-01-17

**Authors:** Abdulhakeem A. Ajadi, Nadia M. Shuaib, Adel F. Shoukry

**Affiliations:** Chemistry Department, Faculty of Science, Kuwait University Kuwait a.shoukry@ku.edu.kw

## Abstract

This research involved constructing and studying plastic membrane Cd(ii)- and Pb(ii)-ion selective electrodes of the coated wire type based on nanoparticles of CdS and PbS as ionophores, respectively. The electrodes exhibited average linear concentration ranges of 1.0 × 10^−6^ to 1.0 × 10^−2^ and 9.6 × 10^−7^ to 1.0 × 10^−2^ M, average detection limits of 8.6 × 10^−7^ and 5.8 × 10^−7^ M, pH ranges of 2.2–5.8 and 2.9–5.9, and average calibration graph slopes of 28.56 and 28.81 mV per concentration decade, respectively. Both electrodes showed high selectivity towards many inorganic cations. Depth profiling X-ray photoelectron spectroscopy of fresh and expired membranes proved that: (a) the nanoparticles were homogeneously dissolved in the polymeric network and (b) the limitation of the life span of the plastic membrane was due to leaching of the active ingredient from the membrane surface to the bathing solution. The topography of fresh, active, and expired membranes as imaged by atomic force microscopy revealed the formation of a gel layer at the surface of the active electrode and drastic deformation of the expired membrane's surface.

## Abstract 2

Nanoparticles of CdS and PbS as ionophres for plastic membrane metal cations electrodes.

## Graphical abstract

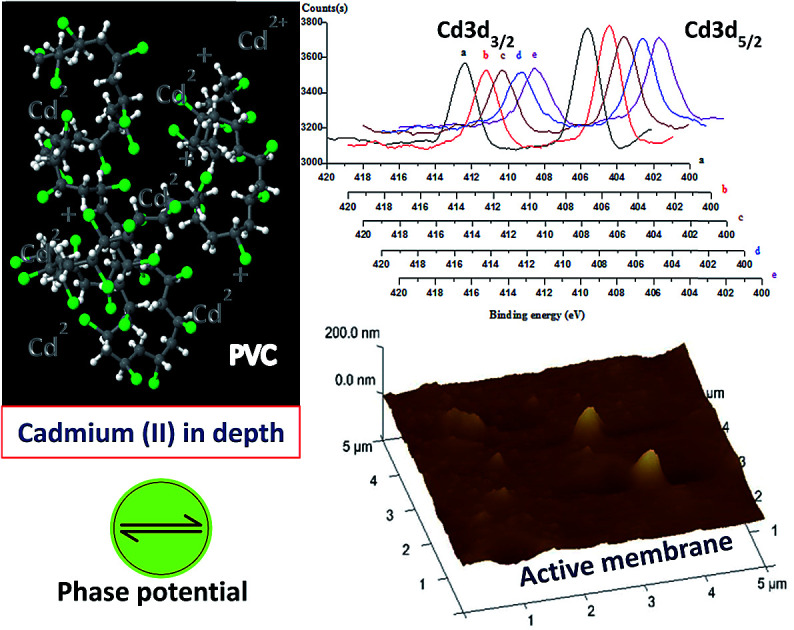

## Introduction

Environmental pollution from Cd(ii) and Pb(ii) has been widely acknowledged as a significant human health threat. Among the diverse techniques used to determine these pollutants, potentiometry using ion-selective electrodes has proven to be cost effective, simple, precise, and reliable.^1,2^

Coated-wire plastic membrane ion-selective electrodes with solid contact have been a popular generation of potentiometric sensors, because of their ease of miniaturization and the possibility of constructing them in micro size, in addition to their wide applications.^3^ The electrode is an electronic conductor contacted directly to the electrode body (the electronic conductor) or *via* another electroactive transducer, which overcomes the ill-defined contact between the electronic conductor and the plastic membrane inner surface.^4^ In a previous work,^5^ it was found that using CuS as the ionic transducer between a diphenhydramine-responsive plastic membrane and the copper electrode body stabilized potential readings and substantially increased the electrode's signal-to-noise ratio. Many conductive and non-conductive epoxy resins have been employed to construct ion-selective electrodes. The function of the resin in these electrodes was to glue the membrane in conventional-type electrodes, or to mount the crystalline membrane at a solid-membrane electrode.^6^ A commercial Araldite M paste mixed with graphite powder was used as a supporting conductor, electrode body, in a coated wire selective electrode for Cu(ii).^7^ In the present work, a film of a conductive silver epoxy resin was tried as a contact between the copper electrode body and the metal-responsive plastic membrane.

Bulk transition metal sulfides mixed with silver sulfide, as an ionic conductor, were used as ionophores in the construction of solid membrane electrodes for some metal cations.^8,9^ These ion exchangers were very successful and have been used in the manufacture of several commercial metal potentiometric sensors.^10^ However, it has been impossible to prepare electrodes with polymeric membranes containing bulk metal sulfides. This was due to the difficulty of dissolving bulky sulfide molecules in the network of the polymeric membrane. Due to their size and the effect of confinement on the particles' lipophilicity, nano-sized metal sulfides, in contrast to bulk sulfides, can be dissolved in the plastic membrane to construct metal cation-selective electrodes.^11,12^ Song *et al.*^13^ prepared plastic membrane Pb^2+^-selective electrode using nano-PbS particles synthesized by the phase transfer method. Nevertheless, the electrode showed a narrow working concentration range of about 10^−4^ to 10^−2^ M and relatively poor selectivity towards the monovalent cations, Na^+^ and K^+^. This may be attributed to non-optimized membrane composition and/or electrode constituents. In the present work, Pb(ii)- and Cd(ii)-plastic membrane electrodes based on nanoparticles of PbS and CdS were developed and their performance characteristics were examined.

In general, the present electrodes showed comparable, and in some cases better performance characteristics over the recently published corresponding electrodes. For example, 3-acetylsemicarbazone ligand has been used^14^ as ionophore in Cd(ii)-selective electrode based on PVC membrane plasticized with dioctylphthalate. The electrode showed Nernstian response over the concentration range 1.0 × 10^−5^ to 1.0 × 10^−1^. However, the electrode life span was limited to five weeks only. Polyaniline Sn(iv) composite cation exchange membrane has shown potentiometric response to Cd(ii) over the PH range 3.5–6.5 and lower detection limit down to 1 × 10^−7^ M. The sensor was used as indicator electrode for titrating Cd(ii) with EDTA standard solution.^15^ Jasinski *et al.*^16^ used 25,26,27,28-tetraki(piperidinylthiocarbonylmethylene)-*p-tert*-butyl calcin[4] as ionophore for Pb(ii)-electrode. The sensor showed Nernstian performance; nevertheless it was selective for Cd(ii) towards only Na(i), Cu(ii), Zn(ii), Mg(ii) and Ca(ii). Pb(ii)-electrode based on 1,2-bis(*N*-benzoylthioureido)benzene; as Pb(ii)-ionophore exhibited Nernstian response for Pb(ii) over the concentration range 6.3 × 10^−8^ to 3.9 × 10^−2^ M and detection limit of 2.5 × 10^−8^ M.^17^ However, the ionophore showed cability for complexation with some cations other than Cd(ii). A solid contact Pb(ii)-electrode has been recently constructed^18^ based on electrospun polyaniline microfibers as the ion-to-electron transducer. The electrode exhibited Nernstian response over the concentration range 10^−9^ to 10^−3^ M, and a detection limit of 6.3 × 10^−10^ M. The electrode showed deviation for concentration of Pb(ii) higher than 10^−3^ M.

The limited lifetime span of plastic membrane electrodes is a general drawback of all plastic membrane potentiometric sensors. Very little has been reported about the grounds of this phenomenon. Previous studies on pharmaceutical cations-plastic membrane electrodes^19–21^ revealed that the electrodes' limited lifetime span is an effect of leaching of the ion exchangers from the membrane surface into the test solution, in addition to deformation of the gel layer. In the present study, surface analyses using depth-profiling X-ray photoelectron spectroscopy (XPS) and atomic force microscopy imaging were applied to surfaces of fresh, active, and exhausted electrodes to reveal the reason(s) for the membrane's limited lifetime span in the case of nanoparticles-based electrodes.

## Experimental

### Chemicals and materials

All reagents used were of analytical grade. The nanoparticles of CdS and PbS were prepared as previously described.^22^ Polyvinyl chloride (PVC) of high molecular weight (Fluka), dioctyl phthalate (DOP) (Sigma-Aldrich), and tetrahydrofuran (Fluka) were used to conduct the potentiometric study of the Cd(ii) ion-selective electrode. The conducting silver epoxy resin had been obtained from MG Chemicals, Canada (8331-silver conductive epoxy). Bi-distilled water was used to prepare the reagents and solutions.

### Electrode preparation and potential measurements

Pure copper rod of 1.0 cm diameter and 12 cm length has been insulated from the air by tight polyethylene tube leaving about 1.0 mm at one end for coating and 0.50 cm at the other end for connection. The polished copper surface has been coated with the conducting epoxy resin and allowed to dry in air overnight. The surface was then coated with the active membrane by spreading slurry consisting of 1.0–4.0% of nano CdS or PbS, 33% of PVC, and 66–63% (w/w) of DOP, dissolved in the least amount of THF, over the surface of the conducting silver epoxy resin using a glass spatula. The membrane formed was left to dry in the air for approximately 2 minutes, and so the operation was iterated until a plastic membrane of about 1.0 mm thickness was formed. The prepared Cd(ii)- and Pb(ii)-electrodes were conditioned by soaking in 1.0 × 10^−3^ M Cd(NO_3_)_2_ or Pb(NO_3_)_2_ solution, respectively, for 24 hours. Measurements had been conducted with a HANA pH/mV meter, Model HI2216, Romania. The following electrochemical system was used:Ag|AgCl|Cl^−^(3M)|Cd^2+^ or Pb^2+^|CdS or PbS|Ag-epoxy resin|Cu

### Depth profiling X-ray photelectron spectroscopy

XPS spectra were recorded on a THERMO spectrometer, model ESCALAB250 Xi, using AlKα radiation (1485.3 eV). The spectra acquisition and processing had been run out by means of Advantage V5.956. Plastic membrane was carefully cut out from the electrode, and introduced into the preparation chamber with the sample holder, and then degassed until good chamber where the pressure is reduced to 10^−9^ to 10^−10^ Torr. The analysis has been carried out with the parameters: spot size 850 μm, step size 0.1 eV, Dwell time 50 ms, and pass energy of 20 eV. All binding energy values were determined with respect to C 1s line (284.6 eV) originating from adventitious carbon. Depth profiling has been done with an Ar ion gun with 3000 eV, 2.5 mm energy spot size and 20 μA current.

### Atomic force microscopy (AFM)

High resolution atomic force microscopy images for a freshly prepared, activated and expired plastic membranes of the Cd(ii) or Pb(ii)-electrode were obtained utilizing a scanning probe microscope (model RTESP, from Veeco Instruments, USA), provided with tapping mode tips. Elasticity measurements were conducted using a phosphorus-doped Si tip (pyramidal shape) with a tip curvature radius of 3.5–4.5 μm, a spring constant of 20–80 Nm^−1^ , and a resonant frequency nominal of 278–311 kHz (Nano Devices, Veeco Metrology, Sant Barbra, California).

## Results and discussion

### Membrane composition

Soaking the electrode is essential to form a hydrated gel layer at its surface. The membrane's Nernstian response requires an optimum composition that allows thermodynamic favorable phase exchange across the test solution–gel layer interface. This means that in the membrane with optimum composition, the transportation process of the metal cation between the solution and the gel layer under the effect of chemical separation is associated with a decrease in the free energy of the system. Four PVC membranes containing different percentages of nano CdS or PbS were tested. Each of the four compositions contained 33% (w/w) of PVC, and 66% of DOP, in addition to 1.0, 2.0, 3.0, or 4.0% of CdS or PbS nanoparticles. The results show that the optimum membrane composition in both the Cd(ii)- and Pb(ii)-electrodes is 2.0% metal sulfide, 33% PVC, and 65% (w/w) DOP. The two electrodes' responses with this composition were nearly Nernstian after a soaking time of 24 h in a 1.0 × 10^−3^ M metal nitrate salt solution (Table 1). Therefore, these electrodes have been considered for investigating the effect of soaking time on the electrodes' performance, as well as to study other performance characteristics.

**Table 1:** Linear concentration ranges, detection limits, and calibration graph slopes of the Pb(ii)- and Cd(ii)-selective electrodes after different intervals of working time

Electrode	Soaking time (day)	Linear range *M*	Detection limit, *M*	Slope mV per conc. decade
Cd(ii)	1	8.13 × 10^−7^ to 1.00 × 10^−2^	5.01 × 10^−7^	26.1
	5	8.91 × 10^−7^ to 1.00 × 10^−2^	6.31 × 10^−7^	28.0
	18	1.00 × 10^−6^ to 1.00 × 10^−2^	7.58 × 10^−7^	29.5
	26	1.00 × 10^−6^ to 1.00 × 10^−2^	7.94 × 10^−7^	32.5
	33	1.12 × 10^−6^ to 1.00 × 10^−2^	7.50 × 10^−7^	33.5
	42	9.12 × 10^−7^ to 1.00 × 10^−2^	17.8 × 10^−7^	33.5
	48	1.58 × 10^−6^ to 1.00 × 10^−2^	13.2 × 10^−7^	29.0
	56	1.00 × 10^−6^ to 1.00 × 10^−2^	7.50 × 10^−7^	23.0
	70	8.30 × 10^−7^ to 1.00 × 10^−2^	5.01 × 10^−7^	22.0
Pb(ii)	1	1.00 × 10^−6^ to 1.00 × 10^−2^	6.31 × 10^−7^	26.0
	2	8.91 × 10^−7^ to 1.00 × 10^−2^	7.41 × 10^−7^	29.0
	8	1.00 × 10^−6^ to 1.00 × 10^−2^	6.31 × 10^−7^	26.0
	14	1.12 × 10^−6^ to 1.00 × 10^−2^	7.55 × 10^−7^	33.5
	22	1.00 × 10^−6^ to 1.00 × 10^−2^	7.50 × 10^−7^	28.0
	29	1.00 × 10^−6^ to 1.00 × 10^−2^	3.16 × 10^−7^	28.0
	36	8.51 × 10^−7^ to 1.00 × 10^−2^	4.22 × 10^−7^	33.0
	44	7.94 × 10^−7^ to 1.00 × 10^−2^	3.98 × 10^−7^	27.0

### Life span

**Fig. 1:**
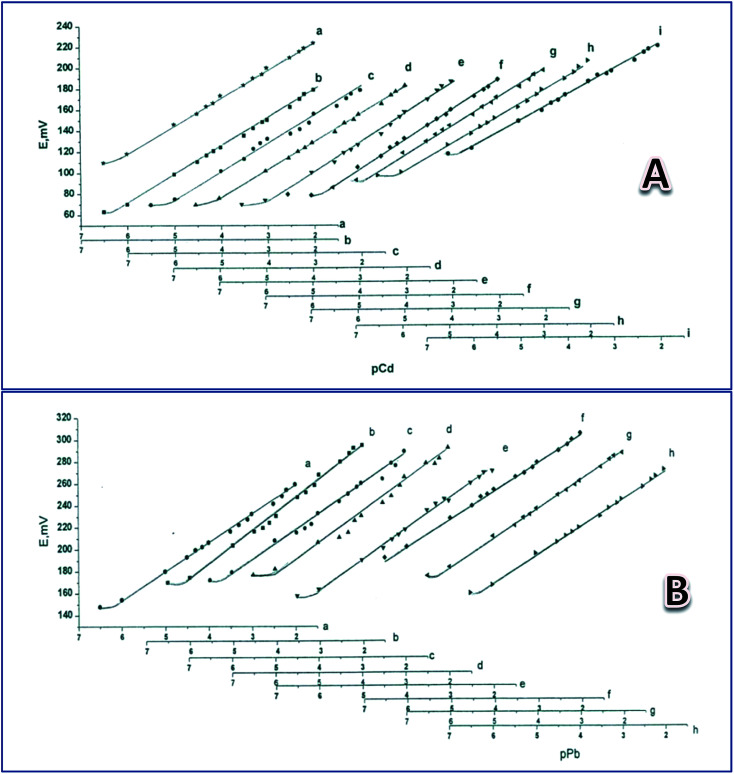
Calibration graphs obtained after soaking the Cd(ii) electrode (A) in 1.0 × 10^−3^ M Cd(NO_3_)_2_ solution for 1 (a), 5 (b), 18 (c), 26 (d), 33 (e), 42 (f), 48 (g), 56 (h), and 70 days (i); and the Pb(ii) electrode (B) in 1.00 × 10^−3^ M Pb(NO_3_)_2_ solution for 1 (a), 2 (b), 8 (c), 14 (d) 22 (e), 29 (f), 36 (g), and 44 days (h).

To study the electrodes' life span when used as Cd(ii)- and Pb(ii)-sensors, they were tested after soaking in a 1.00 × 10^−3^ M solution of the corresponding metal nitrate for different times, as shown in Table 1. Average responses were 28.8 and 28.6 mV per concentration decade towards Pb(ii) and Cd(ii) for 44 and 70 days, respectively (Table 1) (Fig. 1). The average detection limits were 8.65 × 10^−7^ and 5.81 × 10^−7^ M for the Cd(ii) and Pb(ii) electrodes, respectively (Table 1). The Cd(ii)-responsive electrode showed a gradual decrease in calibration graph slope from 29 mV per concentration decade, after 48 days of continuous soaking in Cd(NO_3_)_2_, to 22 mV per concentration grade after 70 days; after that the response decreased to below 20 mV per concentration decade. However, for the Pb(ii) electrode, the calibration graph slope was 27 mV per concentration decade after 44 days of continuous soaking in Pb(NO_3_)_2_, then quickly decreased to below 22 mV per concentration grade after 50 days.

### Effect of pH

**Fig. 2:**
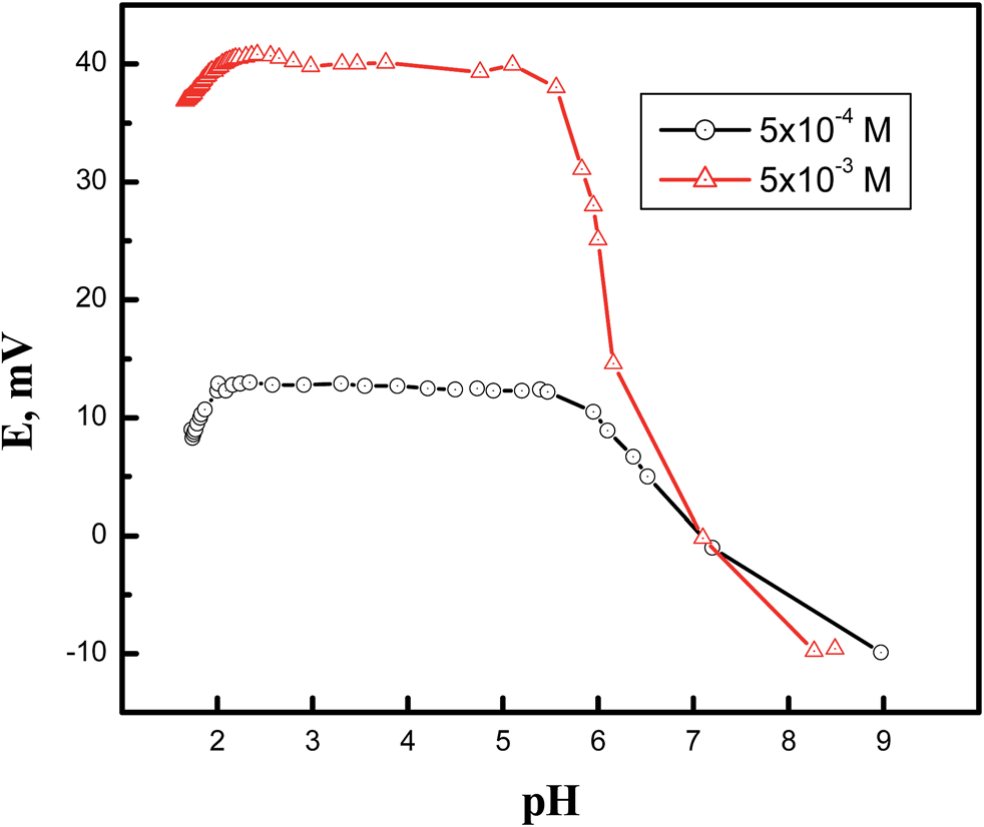
Effect of pH on the potential reading of the Cd(ii) selective electrode.

Fig. 2 demonstrates the effect of pH of the solution (5.0 × 10^−4^ and 5.0 × 10^−3^ M), over the range of 2.0 to 9.0, on the potential reading of the Cd(ii)-selective electrode. Solutions of HNO_3_ and NaOH were used to alter the pH of the solution. The results indicate that electrode potential is not affected by pH changes within an average pH range of 2.24–5.39. It is most plausible that the decrease in the potential reading at pH values higher than 5.39 was due to metal hydration; below pH 2.24, it is probable that the decrease in potential reading was due to competition of the hydronium ions, which existed in a relatively high concentration, and the metal cations for the phase exchange equilibrium at the gel layer of the membrane surface. Similar behavior was noted in the PbS-based electrode, where the electrode showed a working pH range of 2.85–5.92.

### Selectivity of the electrodes

The selectivity coefficient (*K*^pot^_A,J_) of an electrode is a quantitative measure of how it responds to the primary ion (A) in the presence of an interfering ion (J). In the present study, the selectivity coefficient values were determined by the matched potential method first proposed by Gadzekpo and Christian^23^ and reported in the 1995 IUPAC recommendation.^24^ The primary advantage of this method is that it does not need a Nernstian response of the interfering ion. A standard 1.0 × 10^−3^ M solution of the metal nitrate salt was divided into two equal portions. 20 ml of 0.1 M solution of the interfering ion was added to one of the two portions, and then the resultant change in potential was recorded. To the second part of the standard 1.0 × 10^−3^ M solution, 0.1 M metal nitrate solution was added drop wise till the potential reading attained the same value as in the case of the interfering ion. The selectivity coefficients, *K*^pot^_Cd,J_ and *K*^pot^_Pb,J_ (Table 2), were calculated as the ratio of the change of activity of the metal cation to the activity of the interfering ion. It was clear that in all instances, the values of selectivity coefficients (Table 2) were far less than unity, indicating high selectivity of the electrodes for their corresponding ions towards the investigated inorganic cations.

**Table 2:** Selectivity coefficients of the electrodes

Cation	*K* ^pot^_Pb,J_	*K* ^pot^_Cd,J_	Cation	*K* ^pot^_Pb,J_	*K* ^pot^_Cd,J_
Na^+^	8.10 × 10^−2^	2.80 × 10^−3^	Fe^2+^	1.15 × 10^−4^	1.35 × 10^−2^
K^+^	8.70 × 10^−2^	2.13 × 10^−3^	Co^2+^	1.00 × 10^−4^	1.00 × 10^−2^
Ag^+^	1.32 × 10^−4^	1.07 × 10^−2^	Ni^2+^	8.91 × 10^−5^	8.13 × 10^−3^
NH_4_^+^	1.12 × 10^−2^	5.38 × 10^−3^	Cu^2+^	8.32 × 10^−5^	6.61 × 10^−3^
Mg^2+^	3.89 × 10^−4^	6.30 × 10^−5^	Zn^2+^	7.08 × 10^−5^	4.90 × 10^−3^
Ca^2+^	1.29 × 10^−4^	3.16 × 10^−5^	Hg^2+^	1.48 × 10^−5^	4.47 × 10^−6^
Ba^2+^	1.20 × 10^−4^	1.23 × 10^−4^	Al^3+^	257 × 10^−3^	3.02 × 10^−3^
Sr^2+^	1.10 × 10-4	1.27 × 10^−4^	Cr^3+^	7.94 × 10^−3^	7.24 × 10^−3^
Mn^2+^	1.38 × 10^−4^	1.64 × 10^−2^	Fe^3+^	5.37 × 10^−5^	5.37 × 10^−3^

### Depth profiling XPS of the membrane's surface

**Fig. 3:**
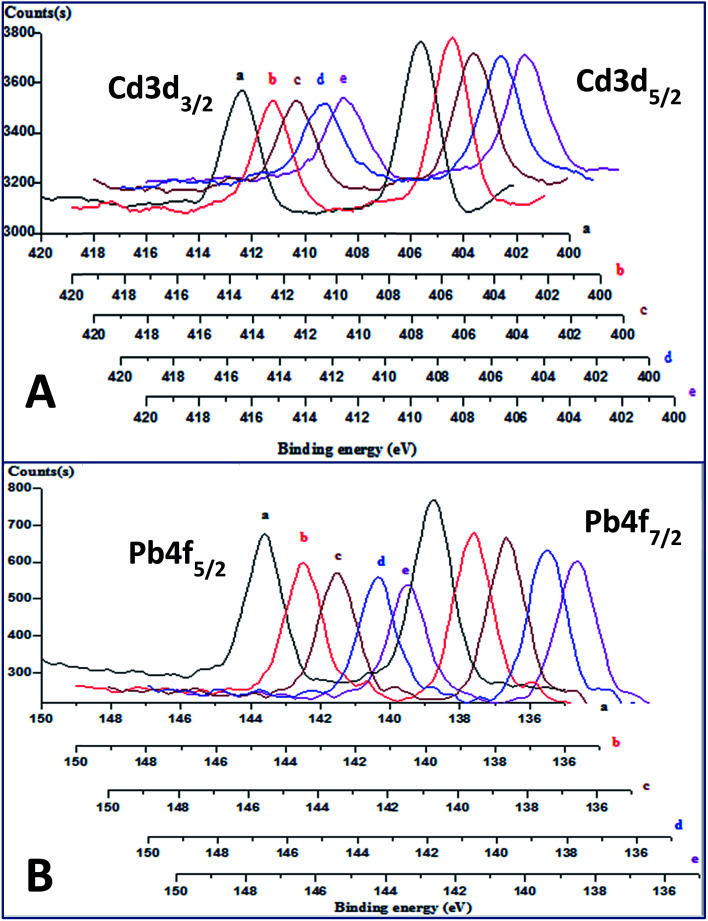
Depth profiling XPS spectra of fresh Cd(ii)-(A), and Pb(ii)-(B) responsive membranes, after etching time of 0 (a), 180 (b), 360 (c), 540 (d), and 720 (e) sec.

The depth proofing spectra of the freshly-prepared CdS-containing membrane (Fig. 3a) for electrons ejected from the core orbit 3d, of spin–orbit couplings 5/2 and 3/2, showed two peaks at 405.7 eV and 412.4 eV, respectively.^25,26^ The corresponding spectra for the PbS-containing membrane showed two peaks at binding energy values of 139.0 and 143.9 eV (Fig. 3b), which were assigned to the ejection of the Pb 4f_7/2_ and Pb 4f_5/2_ core electrons, respectively.^27,28^ The absence of a significant chemical shift in both spectra, at different depths indicates that the nano-sulfide particles in the membranes did not chemically interact with the polymeric matrices. Additionally, the absence of extrinsic satellites in the spectra at higher binding energy values reveals the homogeneous dissolution of the colloidal metal sulfide nanoparticles in the polymeric network of the plasticized membranes.

The performance of the prepared Cd(ii)- and Pb(ii)- electrodes gradually decreased after soaking them for longer periods than 70 and 44 days, respectively. This limitation of the lifetime is a general drawback in almost all potentiometric sensors with plastic membrane type electrodes. To investigate the cause of this limitation, depth profiling X-ray photoelectron spectra was obtained for freshly-prepared and expired Cd(ii)-electrodes (Table 3). The results show that Cd 3d and S 2p peak areas and atomic percentages for the expired membranes were in general less than the corresponding values for the fresh membranes. This indicates leaching of the CdS nanoparticles from the membrane to the bathing solution as a result of the soaking process. These results accord with those of a previous study^14^ on a hydralazinium-selective electrode. It is worth noting that for both membranes, the content of Cd at the surface was higher than in-depth, which is attributed to penetration of Cd(ii) ions from the bathing solution into the gel layer of the membrane surface during the conditioning and working time. Furthermore, the atomic percentages of oxygen at the surface of both membranes were higher than in-depth. This is most plausible due to a gain of oxygen through the membrane uptake of water for hydration during the constitution of the gel layer. The transportation of silicate ions from the Ag-epoxy resin into the membrane's polymeric network is likewise shown by the existence of the Si 2p peaks in the spectra. Similar results have been obtained in case of Pb(ii)-electrode.

**Table 3:** Quantification of X-ray photoelectron spectra of the surface layers of fresh and expired Cd(ii) ion selective electrode based on nanoparticles of CdS

	Time of sputtering, second	C 1s atomic%	O 1s atomic%	Cd 3d atomic%	S 2p atomic%	Cl 2p atomic%	Si 2p atomic%
Fresh	0.00	74.4283	16.8175	0.172445	0.0697382	5.34976	3.162290
	180	88.6130	5.54195	0.133509	0.0927160	4.76829	0.850473
	360	88.1689	5.35846	0.115938	0.0750541	5.52856	0.753072
	540	88.4114	5.14107	0.103730	0.0726562	5.65149	0.619647
	720	88.8022	4.92459	0.109653	0.0628217	5.55016	0.550602
Expired	0.00	77.3921	17.0950	0.0709112	0.0290329	3.11172	2.301250
	180	90.9290	5.23461	0.0224326	0.0490592	3.37226	0.392601
	360	90.6706	4.77166	0.0264361	0.0682116	3.89476	0.568330
	540	90.5997	4.69845	0.0274245	0.0790867	3.88159	0.713796
	720	90.5705	4.65596	0.0335922	0.0877739	3.93821	0.713935

### Atomic force microscopy

**Fig. 4:**
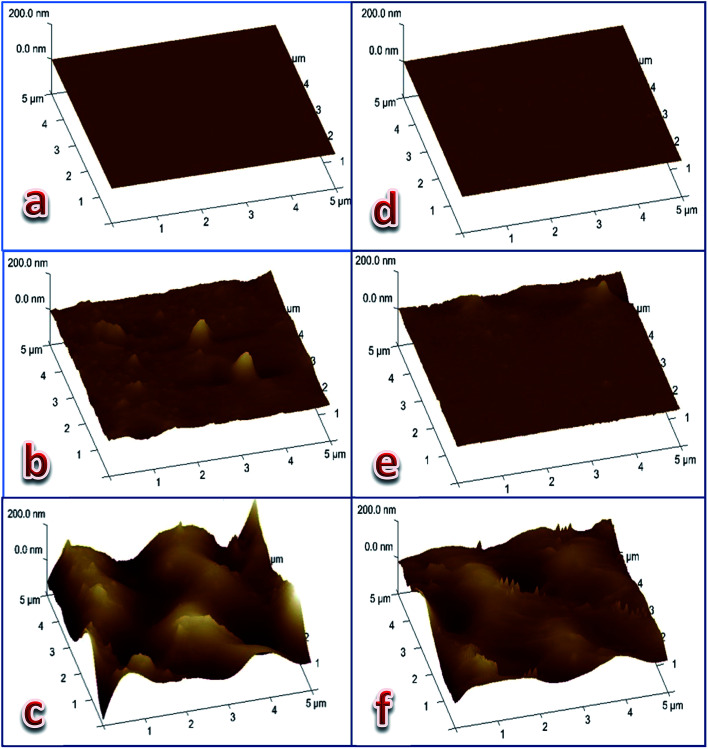
Three dimension (5 μm × 5 μm × 200 nm) AFM images of Cd(ii) electrode (a–c) and Pb(ii)-electrode (d–f). (a, d) Freshly prepared membranes. (b, e) Active membranes. (c, f) Expired membranes.

Three dimensional (5 μm × 5 μm × 200 nm) AFM images of the three membranes' surfaces were taken (Fig. 4) from each of the Cd(ii)- and Pb(ii)-responsive electrodes. The first membrane was a freshly-prepared membrane (of optimum composition), the second was an active membrane preconditioned by soaking the electrode in 1.0 × 10^−3^ M metal nitrate solution for 24 hours, while the third was an expired membrane that was put into work for 80 or 50 days for the Cd(ii)- and Pb(ii)-electrodes, respectively. The images of the membrane surfaces revealed that the smooth surface of the fresh membrane (Fig. 4a and d) was affected by the formation of the gel layer during the activation of the electrode (Fig. 4b and e). The images of the exhausted membrane surface (Fig. 4c and f) clearly show that the electrodes lost their response to the corresponding metal cation as a result of drastic deformation of the surface. This was most likely due to leaching of the ionophore into the bathing solution and partial degradation of the polymeric network. These results are confirmed by the roughness parameters of the surfaces of the three membranes given in Table 4 and agree with previously-obtained results for Pb(ii)-^29^ and Cu(ii)-^30^ electrodes based on coordination compounds as ionophores.

**Table 4:** Roughness parameters of freshly prepared, activated, and expired membranes of the metal cation-selective electrode

Metal cation	Membrane	Working time	*R*_MS_ a (nm)	*R*_a_ b (nm)	*R*_q_ c (nm)	*R*_max_ d (nm)
Cd(ii)	Fresh	Fresh	0.184	0.598	0.712	7.08
	Active	24 hours	1.33	7.57	12.4	160
	Expired	80 days	37.5	41.5	50.0	387
Pb(ii)	Fresh	Fresh	0.184	0.616	0.904	39.4
	Active	24 hours	4.62	3.65	6.58	146
	Expired	50 days	32.7	20.6	26.5	218

a Root mean square.

b Roughness average.

c Root mean roughness.

d The maximum roughness depth.

## Conclusion

Ion-selective electrodes for Cd(ii) and Pb(ii) based on nano-metal sulfide particles as ionophores exhibited a Nernstian response to metal cations over an acidic pH range with high selectivity and sensitivity. The lifetimes of the Cd(ii) and Pb(ii) electrodes were limited to 70 and 44 days, respectively. Depth profiling X-ray photoelectron spectroscopy and atomic force microscopy revealed that limitations of the electrodes' lifespan times were due to leaching of the active ingredient into the bathing solution and deformation of the membrane surface.

## Conflicts of interest

There are no conflicts to declare.

## Supplementary Material
